# Extended hinge length of human IgG3-like antibody enhances early antiviral activity of VRC01 in SHIV-infected rhesus macaques

**DOI:** 10.1128/jvi.00754-26

**Published:** 2026-07-22

**Authors:** Rhianna Jones, Cordelia Manickam, Sweta Shrestha, Kyle Kroll, Griffin Woolley, Maurice Bukenya, Derrick D. Goodman, Katharine J. Bar, Justin Pollara, Georgia D. Tomaras, Francois Villinger, Margaret E. Ackerman, R. Keith Reeves

**Affiliations:** 1 Center for Human Systems Immunology, Duke University School of Medicine 12277, Durham, North Carolina, USA; 2 Department of Surgery, Duke University School of Medicine 12277, Durham, North Carolina, USA; 3 Geisel School of Medicine, Dartmouth College 3728, Hanover, New Hampshire, USA; 4 Department of Integrative Immunobiology, Duke University School of Medicine 12277, Durham, North Carolina, USA; 5 Department of Medicine, Perelman School of Medicine, University of Pennsylvania 14640, Philadelphia, Pennsylvania, USA; 6 New Iberia Primate Research Center, University of Louisiana at Lafayette 4365, New Iberia, Louisiana, USA; 7 Thayer School of Engineering, Dartmouth College 145792, Hanover, New Hampshire, USA; St Jude Children's Research Hospital, Memphis, Tennessee, USA

**Keywords:** IgG3, antibody, SHIV, virus decay

## LETTER

Antibody-based therapies hold promise for the treatment of human immunodeficiency virus (HIV-1) infection, and current research in rhesus macaque (RM) models of infection has demonstrated that neutralizing monoclonal antibody (mAb) treatment can bolster immunity and suppress viral replication (1–3). However, improvements in antiviral potency and breadth are needed to help optimize clinical efficacy (4). Among the many strategies for enhancing antiviral mAb activity being pursued, extended antibody hinge length, as exists naturally in the human IgG3 subclass, has been identified as a defining structural element that can be modified to enhance neutralization (5, 6) and Fc-mediated functions (6–11) against HIV *in vitro*. These effects may be due to increased flexibility conferred by hinge extension (12, 13), facilitating both more effective engagement with viral epitopes on target virions and infected cells, as well as with soluble complement cascade factors and Fc receptors on innate immune cells that mediate antibody effector functions. However, limited evaluation of the functional consequence of this structural change has been conducted *in vivo* (14–16). Here, we report the relative antiviral activity of simianized neutralizing mAb VRC01 in native and hinge-extended formats in reducing chronic viremia in simian-HIV (SHIV)-infected rhesus macaques (RMs). Importantly, this study, for the first time, tests a human IgG3-like molecule in the nonhuman primate model as a therapy for SHIV and shows efficacy against any disease state.

**Fig 1:**
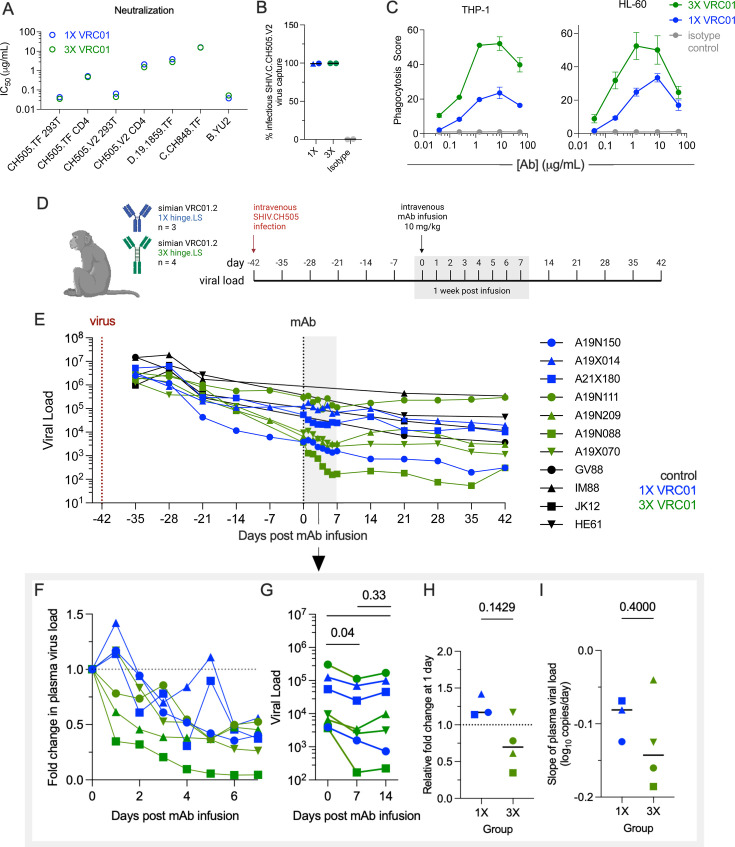
Antiviral activity of VRC01 with modified hinge length. (**A and B**) *In vitro* antibody functions. (**A**) Neutralization potency of simian VRC01.2.LS antibodies with 1X (blue) or 3X (green) hinge compositions. Antibody concentrations at which 50% infectivity (IC_50_) was observed are presented across a panel of SHIV virus sequences and production host cells. (**B**) Percent capture of infectious SHIV CH505 virus by simian VRC01.2.LS antibodies with varied hinge compositions and isotype control. (**C**) Phagocytic activity of simian VRC01.2.LS antibodies with different hinge compositions and an isotype control (gray) in THP-1 monocytes (left) and neutrophil-like DMSO-matured HL-60 cells. Symbol denotes mean, and error bars depict standard error of the mean across triplicate measures. (**D**) Study design of extended hinge broadly neutralizing antibody (simian VRC01.2.LS) impact on plasma viremia over time in SHIV.CH505-infected RMs. (**E**) Viral loads over the entire observation period in the seven animals treated with variant VRC01.2.LS as compared to four untreated controls (black). (**F**) Relative change in daily plasma viral load over the first 7 days after mAb infusion. (**G**) Change in plasma viral load 1 day after treatment (significance by two-tailed Mann-Whitney *U* test). (**H**) Plasma viral load from time of infusion to 1 and 2 weeks post-treatment (significance by Friedman ANOVA with Dunn’s multiple hypothesis correction). (**I**) Linear regression-defined slope of fold change in plasma viral load from day 1 to day 4 post-infusion (significance by two-tailed Mann-Whitney *U* test). Graphical and statistical analyses were performed using GraphPad Prism v10. Panel D was created with BioRender, Ackerman, M. (2026); https://BioRender.com/99ykz6y.

VRC01 was previously simianized to reduce its (17) immunogenicity in RMs, and *in vitro* studies of the antiviral activity of a panel of hinge-extended variants showed that increasing hinge length increased phagocytic activity without affecting affinity for Fc receptors, binding to infected cells, or antibody-dependent cellular cytotoxicity (8). Here, we selected human IgG1-like (1X) and human IgG3-like (3X) hinge variants and further modified them with the M428L/N434S point mutations to increase peak plasma concentration and extend plasma half-life via improved pH-dependent affinity to the neonatal Fc receptor responsible for antibody recycling (17, 18). These hinge variant antibodies exhibited similar neutralizing potency across a panel of SHIV strains, whether produced in cell lines or primary cells (Fig. 1A), and a similar ability to bind infectious SHIV.CH505 virus particles (Fig. 1B). However, the 3X extended hinge antibody showed enhanced phagocytic activity in monocyte-like (THP-1) and neutrophil-like (HL-60) cells, as measured using a previously described phagocytosis assay (19) (Fig. 1C).

To assess whether hinge extension and its associated increase in phagocytic activity improve antiviral activity *in vivo*, a passive transfer experiment was designed to assess the ability of antibody treatment to transiently reduce plasma viral load. Male Indian-origin RMs (*Macaca mulatta*) were intravenously infected with SHIV.CH505 (20), which encodes a clinically relevant primary HIV-1 Env with a closed trimer structure and known sensitivity to CD4bs bnAbs, including VRC01, at a dose of 9,000 infectious units/mL. Forty-two days post-infection, animals received a single intravenous 10 mg/kg dose of simianized VRC01.2 LS antibody (8) with either a human 1X or 3X extended hinge (Fig. 1D). Plasma SHIV RNA levels were determined over time using a two-step real-time qRT-PCR assay (9), and the impact of mAb treatment on the magnitude and kinetics of changes in plasma virus load was investigated.

Prior to antibody infusion, plasma viremia in animals assigned to each mAb treatment group was similar to that observed in untreated historical control (21) animals (Fig. 1E). Following the design of a prior experiment to determine the relative efficacy of mAb interventions to impact the rate and timing of plasma virus clearance (1), systemic virus loads were intensively monitored for a period of 7 days after mAb infusion (Fig. 1F). At 1 week, but not at 2 weeks, after mAb treatment, plasma viremia was significantly reduced (Fig. 1G), despite incorporation of the plasma half-life-extending LS mutation (22). Viral reduction was first observed at different time points across animals, with most (3 of 4) 3X-treated animals showing virus reduction within 1 day of treatment, but no (0 of 3) 1X-treated animals beginning to clear systemic virus during this period (Fig. 1F). In particular, the 3X hinge group showed lower viral loads compared to the 1X group at 24 h of mAb infusion (median viral load fold reduction to 0.7-fold as compared to a 1.2-fold increase over baseline, respectively) (Fig. 1H). To analyze viral decay kinetics during the period after these early fluctuations, we further calculated viral load slopes from 1 to 4 days post-infusion, as previously reported in an experiment of this design (1) and found that the mean rate of decrease in plasma viremia was improved by 1.75-fold when the antibody hinge was extended (Fig. 1I).

Overall, by any measure evaluated, extending mAb hinge length was no less, but potentially more effective in reducing plasma viremia than the typical (1X) hinge length. *In vivo* antiviral activity to reduce plasma viremia was modest in magnitude and only transiently observed, likely due to the small study size; however, the improvements in viral clearance observed here suggest potential value regardless. Future studies evaluating both neutralizing and Fc effector effects of hinge-extended mAb with larger cohorts and testing the *in vivo* efficacy of hinge-engineered mAbs against diverse viral strains are critically needed to define the therapeutic advantages of the extended hinge structure for optimized mAb-based antiretroviral therapy. However, despite limitations, this study, in combination with prior work in RM (1, 15, 23–25) and humanized mouse (5, 14, 16, 26–30) models, shows that antibodies that provide both potent neutralization and robust Fc effector responses can play a role in virus suppression and control.
